# Examining the Relationships among Cognitive Ability, Domain-Specific Self-Concept, and Behavioral Self-Esteem of Gifted Children Aged 5–6 Years: A Cross-Sectional Study

**DOI:** 10.3390/bs11070093

**Published:** 2021-06-22

**Authors:** Dimitrios Papadopoulos

**Affiliations:** 1 Department of Psychology, Gallos University Campus, University of Crete, 74100 Rethymno, Greece; d.papadopoulos@uoc.gr; 2 Department of Psychology, South-West University “Neofit Rilski”, 2700 Blagoevgrad, Bulgaria

**Keywords:** giftedness, cognitive ability, IQ, gifted children, self-esteem, self-concept

## Abstract

Although childhood is a critical period of development during which all children begin a lifelong process of self-discovery that shapes their identities, few studies have focused on the self-concept and self-esteem of young, gifted children. This study recruited 108 gifted children aged 5–6 years from Greece and their preschool teachers to explore the relationships among cognitive ability, domain-specific self-concepts, and global self-esteem. The Pictorial Scale for Perceived Competence and Social Acceptance was used to assess the domain-specific self-concepts of the participants, whereas behavioral manifestations of self-esteem were rated by the children’s teachers using the Behavioral Academic Self-Esteem Scale. There were positive correlations among IQ, perceived scholastic competence, and global self-esteem. Hierarchical regression analysis indicated that significant predictors of global self-esteem were male gender, higher IQ, perceived scholastic competence, and perceived maternal acceptance. Additionally, there were gender differences in global self-esteem and perceived physical competence in favor of boys, whereas perceived maternal acceptance favored girls. This article discusses the need for practitioners working with gifted children to enact a comprehensive social–emotional learning curriculum in schools that promotes academic as well as personal and character strengths. Finally, the limitations of the study and suggestions for future research are also presented.

## 1. Introduction

Childhood is a critical period of development during which both gifted and average children begin the lifelong process of self-discovery that shapes their personality and identity [1]. Harter [2] asserted that children aged between 5 and 7 years develop new cognitive acquisitions that allow them to give vivid descriptions of their virtuosity. Moreover, young children typically begin to describe themselves as having a spectrum of specific competencies that range from academic to physical and social domains (e.g., I am good at football and writing and/or I am not good at swimming and drawing). The positive representation of the child’s competency reinforces the child’s intrinsic desire to actively participate in challenging activities, with the child persisting with the task and aiming for mastery. This mechanism enhances a continuous cycle of pursuit and commitment to improve performance, which in turn influences how the child perceives himself/herself [3,4], and may strengthen the development of other aspects of the self, such as self-confidence and self-esteem [5]. Therefore, developing a positive sense of self throughout one’s early years can aid in acquiring strategies and skills to cope with future challenges [6].

Studies indicate that young children’s self-perceived overall worth as a person cannot be accurately conceptualized because of age-related cognitive and developmental limitations that prevent children from easily verbalizing such concepts [7]. Accordingly, children may have trouble responding symbolically and making sense of abstract statements such as “I’m a valuable person” or “I like to set goals for myself” [8]. However, even at a young age, children can develop a rudimentary sense of self-esteem and may experience low or high self-esteem, which can be identified through behavioral-based manifestations that are observable by adults such as teachers, childcare providers, and parents [7,9]. For example, young children with high self-esteem are typically active, exploratory, and persistent, and they tend to participate fully in daily activities at home and at school, showing mastery, whereas children with low self-esteem tend to react inappropriately to transitions and avoid challenge and exploration [10]. 

In psychology, the notion of the self has been described using interchangeable terms, including self-esteem and self-concept [11]. However, an accurate categorical distinction between these two constructs is not supported by empirical research [12]. Self-esteem, which is generally described as the feelings and appraisals one has regarding oneself in different roles and domains of life [13], includes both emotional and cognitive facets, as does self-concept, which has recently been perceived as a dynamic multidimensional construct [14,15] that is generally described as one’s self-beliefs. From this perspective, both self-esteem and self-concept can be conceptualized as members of the common self-view category [16], which can be examined in association with the meaning individuals attribute to their experiences. 

Research on giftedness supports the idea that gifted children develop their thinking and reasoning abilities earlier than do their non-gifted peers [17], and they can move faster through the curriculum in the area of their talents [18]. Neuropsychological studies suggest that children with high intelligence learn more rapidly than others, possibly due to enhanced frontal cortical activation and faster neural processing speed [19]. This neuronal efficiency promotes the development of high-level neurocognitive capabilities, including enhanced executive functions and an effective working memory system. Gifted children can thereby master a domain (e.g., language, physics, mathematics, arts) easily and rapidly at an early age by teaching themselves, with little or no help from adults [20]. In addition, research has suggested that gifted children tend to develop in-depth understanding of their self-perceptions because of their exceptional abilities [21,22]. This understanding allows gifted individuals to develop great awareness of personal values and morals, which are linked to their well-developed subjective self-representations and unique experiences of the world [23]; further, these experiences differ from those of their average-ability classmates. 

During critical childhood years, global self-esteem is an important personal variable that contributes to academic development and positive school outcomes for both gifted and average-ability students [24]. Coopersmith [10] and Markus and Nurius [25] suggested that positive self-esteem increases children’s confidence, which in turn can contribute to greater cognitive aptitude and success at school [26], with individuals being more able to complete difficult tasks successfully. 

Empirical studies of self-esteem and giftedness have highlighted that self-esteem is a catalyst in the process of talent development [27] and is positively correlated with motivation and superior academic outcomes [28]. However, results of studies of self-esteem in gifted children are varied. Versus their average peers, gifted children have been reported to exhibit higher [29], lower [30], or equivalent [31] self-esteem. However, the current literature suggests that, although gifted children can face complex socio-emotional concerns that threaten their subjective well-being, they are at least as psychosocially well-adjusted as their average-ability classmates [32]. The discrepancies in reported self-esteem among gifted students could have been influenced by various factors. From a developmental perspective, the self-esteem of gifted children can be influenced by social and emotional characteristics, such as emotional intensity, perfectionism, and difficulties in relationships with peers caused by asynchronous development [17]. Silverman [33] (p. 209) reported that “giftedness is a ground of experience that differs significantly from the norm”. Indeed, gifted children who feel different may display low self-esteem, as they lack the skills required to diminish the gap that exists between their intellectual and interest levels and those of their non-gifted peers. Furthermore, the type of giftedness and areas of cognitive strength may also influence this heterogeneity. For example, individuals who are verbally gifted are more vulnerable than those who are mathematically talented [34,35]. 

One of the most important goals of education is to provide opportunities for students to develop and maintain a positive self-concept, which is particularly apparent in educational programs designed for gifted students [36]. Self-concept has been found to be critical in achieving valued personal, social, and academic goals in both gifted and typically developing children [37]. Thus, a positive self-concept experienced by students, particularly in the domain of achievement, has been associated with optimal learning and academic success in school as well as subsequent commitment, motivation, and educational aspirations [38,39]. Additionally, self-concept is positively related to various psychosocial factors, including happiness [40] and greater prosocial behaviors [41]. Furthermore, a strong self-concept is associated with low evaluative anxiety in school, as individuals who feel academically confident are usually successful in test situations, which, in turn, leads to less fear of failure in evaluative situations and therefore greater academic achievement [42]. 

Research evidence is inconsistent regarding gifted children’s self-concept. Some scholars have found that gifted students tend to score higher on academic self-concept, but score lower on physical and social self-concept than their non-gifted peers [34,35,43]. In contrast, other researchers found no difference in self-concept between gifted and typically developing students [44,45], whereas Pyryt and Mendaglio [46] and Bain and Bell [47] found that gifted children scored higher not only on academic, but also on social and physical self-concept. From an alternative perspective, Strop and Goldman [48] found that gifted persons have a low level of general self-concept as a consequence of unrealistic expectations from their teachers and parents, resulting in a sense of anxiety and frustration. These discrepancies suggest that gifted and talented students are a heterogeneous group, as is the nature of their self-concepts; therefore, various profiles can be differentiated [49]. 

Many of these conflicting research results regarding self-concept may be related to the educational setting in which the gifted children were placed. Zeidner and Schleyer [50] conducted a cross-sectional study that examined the academic self-concept, school grades, and test anxiety of 1020 gifted Israeli students attending mixed-ability classes and homogenous special classes for gifted students. They found that participants in the dedicated classes had lower academic self-concept than those who participated in mixed-ability classes. Indeed, gifted students tend to feel more confident about themselves in heterogeneous classrooms, in which they are among the most able, rather than in homogenous ability classes, in which all students have exceptional abilities and outstanding school outcomes [51]. This phenomenon is known as the “Big-Fish-Little-Pond Effect” (BFLPE); it states that able students in high-ability environments have lower academic self-concept than those students with equal abilities in lower-ability settings. That is, being a big fish in a small pond benefits one’s academic self-concept. Research has demonstrated that the BFLPE is likely the result of students comparing their competence to the average ability of their peers [52]. Further, research has shown that gifted children, when placed in segregated classrooms, experience less loneliness [53], have more friends [54], and tend to perceive themselves as acceptable [55]. 

Gifted children’s self-concepts develop from interactions among perceptions of personal experiences and reflected evaluations, as well as from social understanding [56]. Thus, the development of self-concept is impacted by comparisons with peers and the characteristics of the environment in which the individual participates actively [57,58]. From a developmental and social perspective, gifted children mature in the social setting in which they live and interact with other people, including the family, and supportive networks significantly affect the individual’s lifestyle, development of beliefs, and values, which can act as the foundation for the creation of the self [27,59]. For example, the transition from kindergarten to elementary school is followed by a substantial development in self-concept, as cognition and environment engender critical changes in the way the individual consciously thinks and evaluates their accomplishments. Moreover, the quality of the parent–child relationship and a secure attachment with parents tend to play a critical role in the foundation of children’s self-views [60]. This is a reciprocal process, as children with high self-esteem may better internalize positive feedback received from significant others. 

Internationally—including in studies from America and Europe—gifted students are frequently identified by their intelligence quotient (IQ) and school grades; both factors have been considered in the literature as acceptable criteria by which to identify giftedness in children [17,26]. Research has shown that other possible criteria by which to evaluate gifted children are thus ignored, resulting in gifted children being identified and subsequently accepted into gifted education services based on their global IQ scores [61]. Thus, talented students in non-academic areas and gifted students from minority groups, such as children from immigrant parents and children from different cultural contexts, are often overlooked and usually excluded from after-school programs offered to students with high abilities. Specifically in Greece, the general educational level is low, and there is a lack of holistic and scientifically organized provision supervised by the Ministry of Education to identify students with gifts and talents. Additionally, adult society, including teachers of gifted persons, holds stereotypes regarding the gifted group that reflect incorrect beliefs and various myths and fears about elitism in education that remain present in Greek society. In this framework, developing talent in Greece and globally is a challenging and demanding process that requires the promotion of knowledge and sensitization of societies regarding the unique characteristics and needs of gifted individuals. 

Given that gifted and talented persons are often considered to be psychologically resilient and immune to problems related to social and emotional development [62], previous studies focused predominately on the academic needs and achievement of gifted individuals, particularly among intellectually gifted students [63]. However, recent research has highlighted the need to place equal importance both on the academic/cognitive and social–emotional development of gifted students to transform their abilities into talents and, therefore, help such persons to succeed in life [17,58]. Previous research from Greece has predominately explored the cognitive, academic, and motivational profiles of gifted individuals. To the best of the author’s knowledge, only one previous study conducted in Greece has focused on the social and emotional development of gifted children: Papadopoulos [64] investigated the effects of a social–emotional learning program on gifted kindergarten students, finding marked differences before and after the program was conducted in variables related to the self. In addition, this is the first study in Europe to examine gifted children’s self-esteem and self-concept in critical preschool years.

This study explored the relationships between demographic variables, cognitive ability, domain-specific self-concepts, and global self-esteem in 5–6-year-old gifted children. This investigation is important because of the limited empirical knowledge about the self-esteem and self-concept of gifted preschoolers, which may vary depending on the individual’s ability and socio-demographic factors. The hypotheses were that (a) the cognitive ability, perceived scholastic competence, and global self-esteem of 5–6-year-old gifted children would be associated; (b) there would be gender differences in self-concepts and global self-esteem outcomes; and (c) cognitive ability, gender, and domain-specific self-concepts would be significant predictors of the global self-esteem of 5–6-year-old gifted children.

## 2. Materials and Methods

### 2.1. Participants and Procedure 

A cross-sectional survey study was conducted of 108 gifted preschool children (59 boys, 49 girls; age range 5–6 years, M_age_ = 5.52, SD = 1.68) who were attending mixed-ability classes in private kindergartens in the metropolitan area of Athens, and at the time of the study were taking part in an after-school program. To be eligible for the study, each gifted child was required to have a signed report from a licensed psychologist certifying that his/her full-scale IQ score was 120 or above according to the Wechsler Preschool and Primary Scale of Intelligence (WPSSI-III-Greek version). This cutoff score has been proposed by other researchers to identify giftedness and for enrollment in services for gifted persons [17,65]. Participants who had been previously diagnosed with any learning disability or mental health condition were excluded from the study. 

First, the purpose and aims of the study were explained to parents, teachers, and children. Second, parental written consent was obtained, and gifted children agreed to participate in the study verbally. Third, both gifted children and their parents were informed that they could withdraw their participation at any time without penalty. Furthermore, participants were assured that their personal information would be anonymized to protect their confidentiality. All measurements were completed under the same conditions, and the administration of the questionnaires was carried out by the author, who checked that all measurements and parental socio-demographic data were fully completed. The study adhered to the principles outlined in the Declaration of Helsinki. It is important to note that the data for this study were collected by the author as part of a larger research project focused on the self-perceived abilities of gifted children which was supported by the South-West University “Neofit Rilski”. 

### 2.2. Measures

#### 2.2.1. Cognitive Ability

The Wechsler Preschool and Primary Scale of Intelligence (WPPSI-III) [66], Greek version [67], is a cognitive ability measure for children aged 2.6–7.3 years that provides an overall ability score (the Full-Scale IQ) and scores on four factor indices: verbal IQ (VIQ), performance IQ (PIQ), processing speed quotient (PSQ), and a general language composite (GLQ). Wechsler scales are commonly used to identify gifted children and to plan psycho-educational services [65]. Scores are reported as standard scores with a mean of 100 and a standard deviation of 15. WPPSI-III index score reliability coefficients have been reported as high, ranging from 0.72 (for animal coding, a processing speed subtest) to 0.86 (for the Full-Scale IQ).

#### 2.2.2. Self-Concept

The Pictorial Scale of Perceived Competence and Social Acceptance for Young Children [68], Greek version (PATEM-I) [69], was used to evaluate children’s perceived four domain-specific self-concepts: (1) Scholastic competence, (2) Physical competence, (3) Peer acceptance and (4) Maternal acceptance. Each of the four subscales is comprised of five items, constituting a total of 20 items. The instrument is available in two versions, one for girls and one for boys, which minimizes respondents providing socially desirable answers. Specifically, 20 picture pairs are presented to the child, which show a gender-matched child succeeding in an activity and performing less adequately in the same activity. First, participants indicate which image they are “most like”. Second, participants rate whether the image chosen is “really true for me” or “sort of true for me”. Items are scored on a four-point scale (from 4 to 1); then, scores are averaged across the five items for each subscale [69]. Thus, each child receives a score between 4, indicating the highest self-concept, and 1, representing the lowest self-concept. In this study, the internal consistency for self-concept domains ranged from 0.702 for physical competence to 0.814 for scholastic competence. The administration of the instrument required approximately 15–20 min.

#### 2.2.3. Self-Esteem

The Behavioral Academic Self-Esteem Scale (BASE) [9], Greek version [70], consists of 16 sentences that measure global self-esteem in children aged 4 to 16 years. A teacher completes the questionnaire through direct observation of students’ behaviors in the classroom, by grading how often the learner behaves in certain ways. For example, items focus on how well the student fits with the school environment and if he/she displays behaviors that facilitate the learning process (e.g., “This child cooperates with other children”) as well as if he/she makes decisions, participates, or asks questions (e.g., “This child is willing to undertake new tasks”). BASE uses a five-point Likert scale (1 = never, 5 = always); overall self-esteem is calculated as the sum of the scores for each statement. The BASE scale is hand scored and total scores range from 1 to 80, with higher scores indicating higher self-esteem. In the current study, the internal consistency of the BASE as assessed by Cronbach’s alpha was 0.941. 

### 2.3. Data Analysis

Categorical and scale variables were summarized using frequencies, percentages, mean values, and standard deviations (SD). Student’s t-test for independent samples was used to assess significance of differences according to gender. Effect sizes were calculated as Cohen’s *d*. Pearson correlations were used to evaluate the relationships between IQ, domain-specific self-concepts, and global self-esteem. Finally, hierarchical linear regression was used to assess the relationship between the total self-esteem score and demographic variables, IQ, and self-concept domains. In the first step, gender, age, family status, paternal and maternal education, and IQ were included in the regression model. In the second step, perceived scholastic competence was entered. In the final step, after adjusting for sociodemographic variables, IQ, and perceived scholastic competence, the remaining self-concept domain variables were included in the model. All statistical analyses were performed using IBM SPSS Statistics (version 25.0; IBM, Armonk, NY, USA). All statistical tests were two-sided with significance defined as *p* < 0.05.

## 3. Results

More than half (*n* = 59, 53.7%) of the participants were boys and most participants lived with both parents (81.5%). The most frequent level of parental educational attainment for both fathers and mothers was a university/college degree, followed by a master’s or PhD degree. Participants’ intelligence quotient (IQ) ranged from 120 to 138, with a median of 126. Further details of the participants’ socio-demographic characteristics are shown in Table 1.

The participants’ baseline descriptive characteristics for self-concept domains and global self-esteem are presented in Table 2. The vast majority was characterized by a moderate level of self-esteem, followed by high self-esteem. Regarding the self-concept domains, most children were categorized as having a high score on scholastic competence, whereas for physical competence, more than half belonged to the medium score category. Further, participants scored high both in terms of peer and maternal acceptance. 

Pearson correlation coefficients were used to assess the association between cognitive ability and the dependent variables. As illustrated in Table 3, there was a positive relationship between IQ and perceived scholastic competence (*p* < 0.05) and global self-esteem (*p* < 0.05). Similarly, scholastic competence was positively associated with maternal acceptance and global self-esteem (*p* < 0.05). In addition, strong associations between maternal acceptance, physical competence, and global self-esteem were found (*p* < 0.01 in each case). Conversely, the association between perceived maternal acceptance and physical competence was low and negative (*p* < 0.05).

Independent samples *t*-tests were conducted to assess possible differences in the variables according to the participants’ gender (Table 4). For global self-esteem, boys had higher scores than girls, *t* = 5, 969, *p* < 0.001. Boys also scored higher on physical competence than girls, *t* = 11, 458, *p* < 0.001. Antithetically, girls had higher scores than boys on maternal acceptance, *t* = −5.662, *p* < 0.001. Effect sizes were large for global self-esteem (*d* = 1.07) and physical competence (*d* = 2.10), favoring male students, and maternal acceptance (*d* = 1.04), favoring female students. There were no significant differences across the remaining outcomes (all *p*-values >0.05).

Finally, hierarchical regression analysis was conducted to determine the associations between self-esteem and various individual-level factors, including participant demographics, IQ, and domain-specific self-concepts (see Table 5). In the first model, participants’ IQ and demographic variables, including children’s gender and age, family status, and parental education, were significant predictors of self-esteem, which accounted for 28.9% of the variance. Among the control variables, male gender (*p* < 0.01) was positively and significantly associated with total self-esteem scores. In the second model, perceived scholastic competence was a significant predictor of self-esteem score (*p* = 0.003), which increased the variance explained by 5.4%. Finally, the addition of perceived physical competence as well as peer and maternal acceptance revealed a significant relationship with the total self-esteem score, with a total of 40.08% of the variance in self-esteem explained.

## 4. Discussion

This study is the first to explore the factors that influence behavioral self-esteem among gifted preschoolers in Greece. The purpose of this study was to determine whether IQ, self-concept domains, and demographic factors are related to the self-esteem of gifted children aged 5–6 years. The overall results confirmed that male gender, higher IQ, perceived scholastic competence, and maternal acceptance were significantly associated with global self-esteem among the study participants. These findings highlight the significance of these factors in the development of global self-esteem in young, gifted children. 

Considering participants’ baseline descriptive characteristics, the gifted students reported high scholastic competence and good relationships with other people, including peers and mothers. Interestingly, the participants’ ratings indicated more favorable self-perceptions when compared with non-gifted samples from other studies both in Greece [69] and internationally [68,71,72,73]. This may indicate that well-developed self-concept domains can contribute to how well gifted children attain their potential [74] and might be protective factors in their social and emotional development [75]. Previous studies revealed that gifted students tend to have higher levels of academic [76] and social self-concept [35] compared to their average-ability peers. The current findings may be explained by the BFLP effect. According to this model, and given that the study participants attended mixed-ability classes, their perceived scholastic competences and cognitive achievements may have been superior to those of their classmates. This, in turn, likely influenced the development of a stronger sense of competence and confidence. Following a developmental–social framework, the academic success of these students may increase their popularity among their classmates at school, which in turn can affect their acceptance into a peer group. All children have a fundamental psychological need to be a part of a group and to be accepted by their peers, as well as to form and sustain long-term interpersonal relationships. This need is profound during childhood, when one of the greatest challenges is establishing secure friendships [27]. Most of the children in the current study were categorized as having a moderate level of self-esteem, as rated by their teachers. Previous studies [45,77,78] also found that gifted children exhibited satisfactory but not necessarily superior self-esteem scores. However, the findings of this study cannot be directly compared with those of previous research, as very few studies have used teachers’ ratings to evaluate global self-esteem among gifted children at this age. 

The examination of gender differences revealed that male students reported higher levels of perceived physical competence and global self-esteem, whereas female students reported higher levels of maternal acceptance. Consistent with the existing literature [79,80,81], the current study showed that boys had significantly higher scores on perceived physical competence, indicating that they were more active than girls. Apparently, boys tend to play more in their everyday lives with motor activities involving their physical strengths, which may contribute to feeling more comfortable and familiar with sports. Conversely, gifted girls exhibited significantly higher perceived maternal acceptance than gifted boys, indicating that girls experience honest and warm relationships with their mothers, a finding that is consistent with current research [82]. This finding can be attributed to the emergence of social gender roles and a masculine stereotype that favors boys. Accordingly, boys are typically encouraged to adopt an independent and active life-style compared to girls who are pressured by parents and other social agents to engage in passive and dependent behaviors [83]. 

Regarding self-esteem, this study revealed significant gender differences in favor of boys. Consistent with the current findings, cross-cultural studies have also found that boys have a higher level of self-esteem than girls [84,85,86]. From a social perspective, gender differences in self-esteem persist in various countries; however, the extent of these differences varies depending on cultural differences in socioeconomic, sociodemographic, gender equality, and cultural value indices [87]. In this respect, gender differences in the current sample may be attributed to different social roles assigned to males and females throughout Greek society, as well as to the differing parental practices with respect to nurturing boys and girls. 

As expected, the results of the main analysis revealed that male gender was significantly associated with the total self-esteem score, which is a finding that has been observed in studies from different cultures worldwide, independent of Western idiosyncrasies [87,88]. For example, Lord et al. [89] conducted a longitudinal study in which they found that male gender was a significant predictor of global self-worth. Alpert-Gillis and Cornell [90] found that elementary schoolchildren’s self-esteem was related to gender, with boys showing a slight advantage. Similarly, Zeidner and Schleyer [50] suggested that gifted boys who attended mixed-ability elementary classes presented better academic self-esteem than girls of the same age, whereas boys in gifted classes scored higher on both academic and social self-esteem domains. Lazaratou et al. [91] found that among high school students in Greece, boys reported higher overall self-esteem scores than girls. However, studies of the role of gender in self-esteem have provided inconsistent findings. For example, Shi and Li [92] found that both female gender and younger age positively affected the self-esteem of gifted children aged 9 to 13 years in China, independently of age and intellectual status. Other studies found that gender did not strongly influence the developmental trajectory of self-esteem, including in gifted samples in the U.S. [93] and Germany [94]. 

Cognitive ability, as measured by full-scale IQ score, was found to be a significant contributor to self-esteem, such that the higher the IQ, the higher the self-esteem scores of the participants. This finding accords with studies indicating that high cognitive ability increases the probability of academic success, which leads to positive emotions, self-regulatory abilities, and positive appraisals of the self [95]. A study in the U.S. of 12,630 high-school gifted and non-gifted students found that participants with high IQ (above the 95th percentile) had higher self-esteem, showed more interest in school activities, and had a higher level of satisfaction with their education than did participants with lower IQ [96]. Further, Guez Peyre et al. [97] revealed that high IQ was a significant predictor of the self-efficacy of 30,489 elementary students in France, particularly in the academic and self-regulatory domains. Similarly, Jurecska, Lee, Chang, and Sequeira [98] found a significant correlation between increased IQ and self-efficacy scores in elementary school-aged children in the U.S. In general, the literature supports the idea that there is a reciprocal relationship between cognitive ability and social–emotional development [99,100]: The individual’s ability influences motivation, effort, commitment, and academic success, which, in turn, can affect self-worth and subjective well-being. From this perspective, cognitive ability may be considered an essential factor not only for academic excellence [101,102] but also for human behavior and social problem-solving ability; this ability may lead to an increase in the individual’s confidence and psychosocial skills. Conversely, some studies have revealed that intellectual performance does not correlate with self-esteem [103,104]. However, these studies did not include persons from various socio-cultural and economic backgrounds, which considerably reduces the strength of their results. 

Perceived scholastic competence was a significant predictor of global self-esteem, indicating that academic achievement, which is strongly influenced by cognitive ability, is a crucial factor in the positive development of self-esteem. Even when other domain-specific self-concepts (i.e., physical competence and peer acceptance) were included in the prediction model, the gifted preschoolers’ views of their academic abilities significantly influenced their general sense of self-esteem. Consistent with this finding, research has emphasized that all children who perceive themselves as competent in academic areas typically have more interest in school, which in turn contributes to them developing an overall higher level of global self-worth [35,105]. Skaalvik and Hagtvet [106] found that academic self-concept was associated with global self-esteem among Norwegian third graders, and global self-esteem did not predict later achievement. Similarly, Campbell et al. [107] found that perceived scholastic competence was a significant predictor of global self-esteem in African American students from low-income families. Interestingly, studies [108,109] have suggested a bidirectional effect in the relationship between academic competence and global self-esteem, suggesting that general self-esteem both influences and is influenced by perceived academic achievement. The connection between perceived scholastic competence and global self-esteem found in the current study might be interpreted in several ways. First, exceptional children usually have more positive experiences with school-related activities and therefore may have more opportunities to develop a sense of security and confidence [105]. Second, a positive perception of school competence may enable the gifted child to prefer to solve challenging cognitive tasks, which in turn can boost their overall self-worth. Third, teachers may provide more learning opportunities for children with higher scholastic competence. Accordingly, students can increase their self-esteem [110]. 

Maternal acceptance, which refers to the mother–child interaction, significantly influenced the global self-esteem of 5–6-year-old gifted children. This finding suggests that the better the perceived relationship with their mother, the better the children’s self-esteem. Furthermore, the study findings support the notion that mothers have a dominant impact on the social and emotional development of children between the ages of 5 and 6 years. Previous theories and research have highlighted that the quality of the early home environment, especially the provision of appropriate play materials in early childhood, is critical for the development of the child’s personality and self-esteem [10,111]. Indeed, building warm and positive mother–child interactions is critical for establishing attachments with caretakers [112], which, in turn, allows children to build trust, confidence, and favorable self-esteem [113]. In this framework, Bishop and Chace [111] and Bournelli, Makri, and Mylonas (2009) [114] have also suggested that maternal attributes, as well as their role in children’s play, are positively associated with children’s creativity. For gifted individuals, the parent (especially mother)–child relationship has been found to be the most important variable in transforming talent and exceptional ability into achievement [115]. Supportive mothers who encouraged their young, gifted children to ask questions and use their imaginations responded appropriately to their child’s developmental behavior and provided unconditional love and engagement in activities with their children [116,117]. Thus, such parenting practices can promote mother–child bonding, which in turn can increase the child’s overall sense of worth and healthy psychosocial development, while reducing the maternal stress associated with responsibilities and unique challenges of nurturing a precocious child, especially in early childhood [118]. Regarding the mother–child interaction, the correlation analysis revealed an important finding: perceived maternal acceptance did not seem to affect the perceived relationship of the child with his/her peers. There may be several reasons for this. First, children at this stage of development tend to perceive the relationship with their mother as more important than their relationships with their peers, and second, perceived peer acceptance is not as powerful or stable as maternal acceptance [114]. 

From a practical perspective, the findings of the current study suggest there is a need for gifted education professionals to address not only the academic development of gifted students but also to promote character education and emotional literacy, which include personal, social, and emotional competencies [119], with the latter serving as a specific learning variable. For example, the creation of a social and emotional learning curriculum is an appropriate strategy for promoting self-esteem [120], particularly for young, gifted children, as difficulties in social–emotional development are most prominent during early childhood given children’s asynchronous development at that time [17]. In such a curriculum, school psychologists and school counselors would use principles derived from both positive psychology and emotional intelligence to promote overall self-esteem, which could lead students to experience greater happiness and emotional well-being [121,122]. Additionally, educators must be trained in various effective teaching practices that motivate gifted learning as well as promote creative thinking potentials [123,124]. Based on the results of the current study, curricula must respond to gender differences among gifted students. Thus, for example, it is necessary to adjust the activities of gifted education programs so that they are effective in promoting global self-esteem for both male and female students. Finally, educators and parents should recognize that gifted children have the same emotional needs as all other children; they need both love and control, attention and discipline, understanding, and support when they feel stress, fear, or when they are in a bad mood. 

### Strengths, Limitations and Future Research

To the best of the author’s knowledge, this is the first study to explore the relationships among cognitive ability, domain-specific self-concepts, and behaviorally presented self-esteem of gifted children aged 5–6 years. Further, no extant studies are available of children at this stage of development in which teachers’ ratings of behaviorally presented self-esteem were associated with the gifted children’s domain-specific self-concept. The current study’s addition of ratings made by teachers of the gifted children represents a strength. Although previous studies have used teacher nominations or other non-formal criteria (e.g., academic performance or school grades) to determine giftedness, participants in this study were identified as gifted after having taken a formal IQ assessment, which is a globally acceptable identification process for identifying giftedness in children [17]. 

However, this study also has some limitations that must be considered when interpreting the results. First, this was a cross-sectional study of gifted preschoolers attending mixed-ability classes in the metropolitan area of Athens. Therefore, the findings may not be easily generalized to schools that offer gifted services, homogenous classes, or other cultures. Second, domain-specific self-concepts were assessed using measures that were primarily based on students’ own perceptions. Thus, children may have provided more positive than negative responses regarding their self-perceived competence and acceptance by peers and mothers [2]. Third, given that the questionnaires applied in this study have been widely used for students in general, it was not possible to compare the sample of gifted students with other gifted samples. The inclusion of a comparable group, such as gifted students who participated in gifted services or in after-school enrichment programs, would enable better understanding of the self-perceptions and self-esteem of the gifted. As it has been well established that giftedness varies in terms of the domain and level of giftedness, as well as the type of talents, future studies should include a variety of different subpopulations of gifted students. Moreover, further studies could use longitudinal designs to assess whether gifted children’s domain-specific self-concepts are associated with self-esteem, particularly their perceptions regarding mother–child interactions, as well as whether this interaction continues to influence children as they age. 

## 5. Conclusions

The results of this cross-sectional study suggest that increased cognitive ability, male gender, and domain-specific self-concepts, including perceived scholastic abilities and maternal acceptance, were related to the behaviorally presented self-esteem of gifted children aged 5–6 years. In addition, this study revealed gender differences in self-esteem and perceived physical competence in favor of boys, and maternal acceptance in favor of girls. Early childhood is not too early to begin considering the development of self-perceptions because these are an essential aspect of the social lives of children that affect emerging beliefs about the self [125]. Identifying the various factors that influence self-esteem in gifted populations and understanding how these may interact is critical for the holistic development and psychosocial adjustment of such persons. However, to better understand the source and stability of these self-beliefs during childhood, future research is needed to explore the social and emotional experiences associated with giftedness. After considering the limitations of the current study, some practical recommendations remain. For example, professionals (licensed child and school psychologists, as well as educators and school counselors) working with young, gifted children should be able to recognize early signs of giftedness and create developmentally appropriate environments that support the different strengths of students, so that such students are successful and creative in tasks that present a challenge to them [17]. This process could develop students’ talent domains and strengthen their overall self-esteem. Finally, studies of self-concept and self-esteem involving young, gifted children in Greece are lacking. Therefore, additional country-level studies are needed to investigate the social and emotional development of such individuals, given that the socio-cultural context in which children are educated and interact with others can influence the development of the self.

## Figures and Tables

**Table 1:** Baseline socio-demographic characteristics of the 108 participants.

Characteristic	Value
Gender (N, %)	
Boys	59 (53.7)
Girls	49 (45.3)
Age (N, %)	
5–5.5 years	97 (89.8)
5.6–6 years	11 (10.2)
IQ	
Median	126
Min–Max	120–138
Family status (N, %)	
Live with two parents	88 (81.5)
Live with one parent	20 (18.5)
Nationality (N, %)	
Greek	101 (93.5)
American	7 (6.5)
Paternal educational attainment (N, %)	
Secondary education	22 (20.4)
Post-secondary education	18 (16.7)
University/college degree	38 (35.2)
Master’s/PhD	30 (27.7)
Maternal educational attainment (N, %)	
Secondary education	21 (19.5)
Post-secondary education	22 (20.3)
University/college degree	42 (39.0)
Master’s/PhD	23 (21.2)

**Table 2:** Descriptive data for baseline characteristics.

	**N**	**%**
**Global self-esteem**		
Low self-esteem (≤44)	5	4.6
Moderate self-esteem (45–66)	86	79.7
High self-esteem (≥67)	17	15.7
**Self-concept domains**		
*Scholastic competence*		
Low score (1–2)	0	0.0
Medium score (2–3)	12	11.2
High score (3–4)	96	88.8
*Physical competence*		
Low score (1–2)	0	0.0
Medium score (2–3)	64	59.2
High score (3–4)	44	40.8
*Peer acceptance*		
Low score (1–2)	0	0.0
Medium score (2–3)	47	43.5
High score (3–4)	61	56.5
*Maternal acceptance*		
Low score (1–2)	0	0.0
Medium score (2–3)	48	44.5
High score (3–4)	60	55.5

**Table 3:** Pearson’s correlations between cognitive ability (IQ), domain specific self-concepts, and global self-esteem.

	IQ	Scholastic Competence	Peer Acceptance	Physical Competence	Maternal Acceptance	Global Self- Esteem
IQ	1					
Scholastic competence	0.192 *	1				
Peer acceptance	0.033	0.147	1			
Physical competence	0.016	0.077	0.016	1		
Maternal acceptance	−0.039	0.212 *	0.316	−0.198 *	1	
Global self-esteem	0.201 *	0.214 *	0.031	0.508 **	0.306 **	1

Note. * *p* < 0.05, ** *p* < 0.01.

**Table 4:** Means and standard deviations (SD) of continuous variables by gender.

	Boys	Girls		
	Mean (SD)	Mean (SD)	*t*-Statistic (*p*-Value)	Effect Size (Cohen’s *d*)
Global self-esteem	56.06 (7.70)	48.89 (5.38)	5.969 (<0.001) ^a^	1.07
Scholastic competence	3.19 (0.21)	3.22 (0.23)	−0.836 (0.405)	0.15
Physical competence	3.11 (0.30)	2.49 (0.29)	11.458 (<0.001) ^a^	2.10
Peer acceptance	2.96 (0.23)	3.01 (0.27)	−0.978 (0.330)	0.18
Maternal acceptance	2.79 (0.24)	3.05 (0.26)	−5.662 (<0.001) ^a^	1.04

Note. ^a^ *p* < 0.05.

**Table 5:** Hierarchical linear regression for global self-esteem.

		B	*t*	*p*-Value	F Change	R^2^	R^2^ Change
Step 1					7.648	0.289	0.289
	Gender	6.629	5.033	<0.001			
	Age	−3.671	−1.811	0.073			
	IQ	0.028	0.269	0.788			
	Family status	−1.620	−0.994	0.322			
	Paternal education	1.113	1.631	0.106			
	Maternal education	0.499	0.756	0.451			
Step 2					9.143	0.342	0.054
	Gender	7.035	5.500	<0.001			
	Age	−0.061	−0.603	0.548			
	IQ	3.947	2.014	0.046			
	Family status	−1.923	−1.219	0.225			
	Paternal education	0.939	1.419	0.159			
	Maternal education	0.199	0.309	0.758			
	Scholastic competence	8.322	3.024	0.003			
Step 3					4.009	0.408	0.065
	Gender	5.232	2.414	0.017			
	Age	−0.067	−0.676	0.500			
	IQ	4.278	2.247	0.027			
	Family status	−1.502	−0.986	0.326			
	Paternal education	0.457	0.656	0.513			
	Maternal education	0.154	0.235	0.814			
	Scholastic competence	8.616	3.075	0.003			
	Physical competence	−2.508	−1.311	0.193			
	Peer acceptance	1.690	0.710	0.479			
	Maternal acceptance	6.350	2.577	0.011			

## Data Availability

Data are available upon reasonable request.

## References

[1] Erikson E.H. (1963). Childhood and Society.

[2] Harter S., Kernis M.H. (2006). The development of self-esteem. Self-Esteem Issues and Answers: A Sourcebook of Current Perspectives.

[3] Brown G.L., Mangelsdorf S.C., Neff C., Schoppe-Sullivan S.J., Frosch C.A. (2009). Young children’s self-concepts: Associations with child temperament, mothers’ and fathers’ parenting, and triadic family interaction. Merrill Palmer Q..

[4] Shavelson R.J., Hubner J.J., Stanton G.C. (1976). Self-concept: Validation of construct interpretations. Rev. Educ. Res..

[5] Nobre G.C., Valentini N.C. (2019). Self-perception of competence: Concept, changes in childhood, and gender and age-group differences. J. Phys. Educ..

[6] Huang C. (2011). Self-concept and academic achievement: A meta-analysis of longitudinal relations. J. Sch. Psychol..

[7] Haltiwanger J., Harter S. (2019). The behavioral rating scale of presented self-esteem for young children. J. Psychiatry Behav. Sci..

[8] Flavell J.H. (1963). The University Series in Psychology. The Developmental Psychology of Jean Piaget.

[9] Coopersmith S., Gilberts R. (1982). Behavioral Academic Self-Esteem: A Rating Scale.

[10] Coopersmith S. (1981). The Antecedents of Self-Esteem.

[11] Harter S., McInerney D., Marsh H.W., Craven R.G. (2012). New directions in self-development: Resurrecting the I-self. Theory Driving Research: New Wave Perspectives on Self-Processes and Human Development.

[12] Byrne B. (2002). Validating the measurement and structure of self-concept: Snapshots of past, present, and future research. Am. Psychol..

[13] Baumeister R., Campbell J., Krueger J., Vohs K. (2003). Does high self-esteem cause better performance, interpersonal success, happiness, or healthier lifestyles?. Psychol. Sci. Public Interes..

[14] Byrne B.M. (1988). Adolescent self-concept, ability grouping, and social comparison: Reexamining academic track differences in high school. Youth Soc..

[15] Harter S., Leahy R.L. (1985). Competence as a dimension of self-evaluation: Toward a comprehensive model of self-worth. The Development of the Self.

[16] Swann W.B., Chang-Schneider C., McClarty K.L. (2007). Do people’s self-views matter? Self-concept and self-esteem in everyday life. Am. Psychol..

[17] Papadopoulos D. (2020). Psychological framework for gifted children’s cognitive and socio-emotional development: A review of the research literature and implications. J. Educ. Gift. Young Sci..

[18] Reis S.M., Westberg K.L., Kulikowich J., Caillard F., Heébert T., Plucker J., Smist J.M. (1993). Why Not Let High. Ability Students Start School in January? The Curriculum Compacting Study.

[19] Geake J.G., Shavinina L.V. (2009). Neuropsychological characteristics of academic and creative giftedness. International Handbook on Giftedness.

[20] Winner E. (1996). Gifted Children: Myths and Realities.

[21] Coleman L.J., Cross T.L., Cross J.R. (2012). Lived experience, mixed messages, and stigma. Handbook for Counselors Serving Students with Gifts and Talents.

[22] Roeper A., Silverman L., Ambrose D., Cross T. (2009). Giftedness and moral promise. Morality, Ethics, and Gifted Minds.

[23] Silverman L.K. (1994). The moral sensitivity of gifted children and the evolution of society. Roeper Rev..

[24] Marsh H.W., Yeung A.S. (1997). Causal effects of academic self-concept on academic achievement: Structural equation models of longitudinal data. J. Educ. Psychol..

[25] Markus H., Nurius P. (1986). Possible selves. Am. Psychol..

[26] Adams M.J. (1996). Youth in crisis: An examination of adverse risk factors effecting children’s cognitive and behavioral–emotional development, children ages 10–16. Diss. Abstr. Int. Hum. Soc. Sci..

[27] Casino-García A.M., Llopis-Bueno M.J., Llinares-Insa L.I. (2021). Emotional intelligence profiles and self-esteem/self-concept: An analysis of relationships in gifted students. Int. J. Environ. Res. Public Health.

[28] Topçu S., Leana-Tascılar M.Z. (2018). The role of motivation and self-esteem in the academic achievement of Turkish gifted students. Gift Educ. Int..

[29] Sarouphim K.M. (2011). Gifted and non-gifted Lebanese adolescents: Gender differences in self-concept, self-esteem and depression. Int. Educ..

[30] Lea-Wood S.S., Clunies-Ross G. (1995). Self-esteem of gifted adolescent girls in Australian schools. Roeper Rev..

[31] Chiu L.H. (1990). Self-esteem of gifted, normal, and mild mentally handicapped children. Psychol. Sch..

[32] Cross J.R., Cross T.L. (2015). Clinical and mental health issues in counseling the gifted individual. J. Counsel. Dev..

[33] Silverman L.K. (1998). Through the lens of giftedness. Roeper Rev..

[34] Jones T.W. (2013). Equally cursed and blessed: Do gifted and talented children experience poorer mental health and psychological well-being?. Educ. Child. Psychol..

[35] Lee S., Olszewski-Kubilius P., Thomson D.T. (2012). Academically gifted students’ perceived interpersonal competence and peer relationships. Gift Child. Q..

[36] Marsh H.W., Chessor D., Craven R., Roche L. (1995). The effects of gifted and talented programs on academic self-concept: The big fish strikes again. Am. Educ. Res. J..

[37] Hoogeveen L., Van Hell J.G., Verhoeven L. (2009). Self-concept and social status of accelerated and nonaccelerated students in the first 2 years of secondary school in the Netherlands. Gift Child. Q..

[38] Guo J., Nagengast B., Marsh H.W., Kelava A., Gaspard H., Brandt H., Cambria J., Flunger B., Dicke A.-L., Häfner I. (2016). Probing the unique contributions of self-concept, task values, and their interactions using multiple value facets and multiple academic outcomes. AERA Open.

[39] Marsh H.W., Martin A.J. (2011). Academic self-concept and academic achievement: Relations and causal ordering. Br. J. Educ. Psychol..

[40] O’Rourke J., Cooper M., Gray C. (2012). Is being “smart and well behaved” a recipe for happiness in Western Australian primary schools?. Int. J. Psychol. Stud..

[41] Schwarzer R., Fuchs R., Conner M., Norman P. (2009). Self-efficacy and healthy behaviours. Predicting Health Behaviour: Research and Practice in Social Cognition Models.

[42] Greenberg J., Solomon S., Pyszczynski T., Rosenblatt A., Hurling J., Lyon D., Simon L., Pinel E. (1992). Why do people need self-esteem? Converging evidence that self-esteem serves as an anxiety-buffering function. J. Pers. Soc. Psychol..

[43] Brounstein P.J., Holahan W., Dreyden J. (1991). Change in self-concept and attributional styles among academically gifted adolescents. J. Appl. Soc. Psychol..

[44] Ablard K.E. (1997). Self-perceptions and needs as a function of type of academic ability and gender. Roeper Rev..

[45] Tong J., Yewchuk C. (1996). Self-concept and sex-role orientation in gifted high school students. Gift Child. Q..

[46] Pyryt M.C., Mendaglio S. (1994). The multidimensional self-concept: A comparison of gifted and average-ability adolescents. J. Educ. Gift.

[47] Bain S.K., Bell S.M. (2004). Social self-concept, social attributions, and peer relationships in fourth, fifth and sixth graders who are gifted compared to high achievers. Gift Child. Q..

[48] Strop J., Goldman D. (2002). The affective side: Emotional issues of twice-exceptional students. Underst Our Gift.

[49] Villatte A., Hugon M., de Léonardis M. (2011). Forms of self-concept in gifted high school students enrolled in heterogeneous classes. Eur. J. Psychol. Educ..

[50] Zeidner M., Schleyer E.J. (1999). The big-fish–little-pond effect for academic self-concept, test anxiety and school grades in gifted children. Contemp. Educ. Psychol..

[51] Marsh H.W., Parker J.W. (1984). Determinants of student self-concept: Is it better to be a relatively large fish in a small pond even if you don’t learn to swim as well?. J. Pers. Soc. Psychol..

[52] Fang J., Huang X., Zhang M., Huang F., Li Z., Yuan Q. (2018). The big-fish-little-pond effect on academic self-concept: A meta-analysis. Front. Psychol..

[53] Cross T.L., Swiatek M.A. (2009). Social coping among academically gifted adolescents in a residential setting: A longitudinal study. Gift Child. Q..

[54] Manor-Bullock R., Look C., Dixon D.N. (1995). Is giftedness socially stigmatizing? The impact of high achievement on social interactions. J. Educ. Gift.

[55] Sayler M.F., Brookshir W.K. (1993). Social, emotional, and behavioral adjustment of accelerated students, students in gifted classes, and regular students in eighth grade. Gift Child. Q..

[56] Rayner S.G., Riding R.J., Rayner S.G. (2001). Aspects of the self as learner: Perception, concept, and esteem. Self-Perception.

[57] Gagné F., Sternberg R.J., Davidson J.E. (2005). From gifts to talents: The DMGT as a developmental model. Conceptions of Giftedness.

[58] Subotnik R.F., Olszewski-Kubilius P., Worrell F.C. (2011). Rethinking giftedness and gifted education: A proposed direction forward based on psychological science. Psychol. Sci. Pub. Interes..

[59] Marsh H.W., Shavelson R.J. (1985). Self-concept: Its multifaceted, hierarchical structure. Educ. Psychol..

[60] Hosogi M. (2012). Importance and usefulness of evaluating self-esteem in children. Biopsychosoc. Med..

[61] Feldhusen J.F., Jarwan F.A., Heller K.A., Mönks F.J., Sternberg R.J., Subotnik R.F. (2000). Identification of gifted and talented youth for educational programs. International Handbook of Giftedness and Talent.

[62] Neihart M., Pfeiffer S.I., Cross T.L. (2015). The Social and Emotional Development of Gifted Children: What do We Know?.

[63] Davis G.A., Rimm S.B. (2004). Education of the Gifted and Talented.

[64] Papadopoulos D. (2020). Effects of a social-emotional learning-based program on self-esteem and self-perception of gifted kindergarten students: A pilot study. J. Educ. Gift Young Sci..

[65] McClain M.C., Pfeifer S. (2012). Identification of gifted students in the United States today: A look at state definitions, policies, and practices. J. Appl. School Psychol..

[66] Wechsler D. (2002). Wechsler Preschool and Primary Scale of Intelligence (WPPSI-III).

[67] Sideridis G., Antoniou F. (2015). Wechsler Preschool and Primary Scale of Intelligence–Third Edition (WPPSI-III GR)—Standardization in Greek.

[68] Harter S., Pike R. (1984). The pictorial scale of perceived competence and social acceptance for young children. Child. Dev..

[69] Makri-Mpotsari E. (2013). How I Perceive My Self I.

[70] Kakouros Ε., Maniadaki Κ., Stalikas A., Triliva S., Roussi P. (2002). Translation and adaptation of the Behavioral Academic Self-Esteem Scale. Psychometric Tools in Greece.

[71] Hoge R.D., Renzulli J.S. (1993). Exploring the link between giftedness and self-concept. Rev. Educ. Res..

[72] Marsh H.W., Plucker J.A., Stocking V.B. (2001). The Self-Description Questionnaire II and gifted students: Another look at Plucker, Taylor, Callahan, and Tomchin’s (1997) “Mirror, mirror on the wall. ” Educ. Psychol. Meas..

[73] Li A.K.F. (1988). Self-perception and motivational orientation in gifted children. Roeper Rev..

[74] Schneider B.H., Schneider B.H. (1987). The social self-concepts of gifted children: Delusions of ungrandeur?. The Gifted Child in Peer Group Perspective.

[75] Neihart M. (1999). The impact of giftedness on psychological well-being: What does the empirical literature say?. Roeper Rev..

[76] Chapman J.W., McAlpine D.D. (1988). Students’ perceptions of ability. Gift Child. Q..

[77] Vialle W., Heaven P.C.L., Ciarrochi J. (2007). On being gifted, but sad and misunderstood: Social, emotional, and academic outcomes of gifted students in the Wollongong Youth Study. Educ. Res. Eval..

[78] Janos P.M., Fung H.C., Robinson N.M. (1985). Self-concept, self-esteem, and peer relations among gifted children who feel “different”. Gift Child. Q..

[79] Cornell D.G., Pelton G.M., Basin L.E., Landrum M., Ramsay S., Cooley M.R., Lynch K.A., Hamrick E. (1990). Self-concept and peer status among gifted program youth. J. Educ. Psychol..

[80] Barnett L.M., Ridgers N.D., Salmon J. (2015). Associations between young children’s perceived and actual ball skill competence and physical activity. J. Sci. Med. Sport.

[81] Afthentopoulou A.-E., Venetsanou F., Zounhia A., Petrogiannis K. (2018). Physical activity, motor competence, and perceived physical competence: What is their relationship in children aged 6–9 years?. Hum. Mov..

[82] Dixon S.K., Kurpius S.E.R., Kerr B. (2009). Academic self-concept. Encyclopedia of Giftedness, Creativity, and Talent.

[83] Bois J.E., Sarrazin P.G., Brustad R.J., Trouilloud D.O., Cury F. (2005). Elementary schoolchildren's perceived competence and physical activity involvement: The influence of parents' role modelling behaviours and perceptions of their child’s competence. Psychol. Sport Exerc..

[84] Swiatek M.A. (2000). Social coping among gifted high school students and its relationship to self-concept. J. Outcome Meas..

[85] Baumeister R.F. (1993). Self-Esteem: The Puzzle of Low Self-Regard.

[86] Chan D.W. (2001). Global and specific self-concepts of gifted adolescents in Hong Kong. J. Educ. Gift.

[87] Bleidorn W., Arslan R.C., Denissen J.J.A., Rentfrow P.J., Gebauer J.E., Potter J., Gosling S.D. (2016). Age and gender differences in self-esteem—a cross-cultural window. J. Pers. Soc. Psychol..

[88] Rudasill K.M., Capper M.R., Foust R.C., Callahan C.M., Albaugh S.B. (2009). Grade and gender differences in gifted students’ self-concepts. J. Educ. Gift.

[89] Lord S.E., Eccles J., McCarthy K.A. (1994). Surviving the junior high school transition: Family processes and self-perceptions as protective and risk factors. J. Early Adolesc..

[90] Alpert-Gillis L.J., Connell J.P. (1989). Gender and sex-role influences on children’s self-esteem. J. Pers..

[91] Lazaratou H., Stavropoulos V., Charbilas D., Soldatou A., Dikeos D. (2015). Self-esteem of Greek adolescents: Changes in a decade of socio-economic hardship. Adolesc. Psychiatry.

[92] Shi J., Li Y., Zhang X. (2008). Self-concept of gifted children aged 9 to 13 years old. J. Educ. Gift.

[93] Orth U., Robins R.W. (2014). The development of self-esteem. Curr. Dir. Psychol. Sci..

[94] Orth U., Maes J., Schmitt M. (2015). Self-esteem development across the life span: A longitudinal study with a large sample from Germany. Dev. Psychol..

[95] Pekrun R., Goetz T., Titz W., Perry R. (2002). Academic emotions in students’ self-regulated learning and achievement: A program of qualitative and quantitative research. Educ. Psychol..

[96] Roznowski M., Hong S., Reith J. (2000). A further look at youth intellectual giftedness and its correlates: Values, interests, performance, and behavior. Intelligence.

[97] Guez A., Peyre H., Le Cam M., Gauvrit N., Ramus F. (2018). Are high-IQ students more at risk of school failure?. Intelligence.

[98] Jurecska D.E., Lee C.E., Chang K.B., Sequeira E. (2011). I am smart, therefore I can: Examining the relationship between IQ and self-efficacy across cultures. Int. J. Adolesc. Med. Health.

[99] Vogl K., Preckel F. (2014). Full-time ability grouping of gifted students: Impacts on social self-concept and school-related attitudes. Gift Child. Q..

[100] Driscoll M.P. (2005). Psychology of Learning and Instruction.

[101] Terman L.M. (1925). Genetic Studies of Genius: Volume 1. Mental and Physical Traits of a Thousand Gifted Children.

[102] Hollingworth L.S. (1942). Children above 180 IQ Stanford-Binet.

[103] Kaya F., Oğurlu Ü. (2015). The relationship among self-esteem, intelligence, and academic achievement. Int. J. Hum. Sci..

[104] Leonardson G.R. (1986). The relationship between self-concept and selected academic and personal factors. Adolescence.

[105] Verschueren K., Marcoen A., Buyck P. (1998). Five-year-olds’ behaviorally presented self-esteem: Relations to self-perceptions and stability across a three-year period. J. Genet. Psychol..

[106] Skaalvik E.M., Hagtvet K.A. (1990). Academic achievement and self-concept: An analysis of causal predominance in a developmental perspective. J. Pers. Soc. Psychol..

[107] Campbell F.A., Pungello E.P., Miller-Johnson S. (2002). The development of perceived scholastic competence and global self-worth in African American adolescents from low-income families: The roles of family factors, early educational intervention, and academic experience. J. Adolesc. Res..

[108] Chohan B.I. (2013). An exploratory study of the relationship between self-esteem and academic performance of the students. J. Educ. Res..

[109] Liu X., Kaplan H.B., Risser W. (1992). Decomposing the reciprocal relationships between academic achievement and general self-esteem. Youth Soc..

[110] Fuchs-Beauchamp K.D. (1996). Preschoolers’ inferred self-esteem: The Behavioral Rating Scale of Presented Self-Esteem in Young Children. J. Genet. Psychol..

[111] Bishop D.W., Chace C.A. (1971). Parental conceptual systems, home play environment, and potential creativity in children. J. Exp. Child. Psychol..

[112] Bowlby J. (1969). Attachment and Loss: Sadness and Depression.

[113] Burns R.B. (1979). The Self-Concept in Theory, Measurement, Development and Behavior.

[114] Bournelli P., Makri A., Mylonas K. (2009). Motor creativity and self-concept. Creat. Res. J..

[115] Olszewski P., Kulieke M., Buescher T. (1987). The influence on the family environment on the development of talent: A literature review. J. Educ. Gift.

[116] Snowden P.L., Christian L.G. (1999). Parenting the young gifted child: Supportive behaviors. Roeper. Rev..

[117] Strom R., Johnson A., Strom S. (1990). Home and school support for gifted children. Int. J. Disabil. Dev. Educ..

[118] Jolly J.L., Matthews M.S. (2012). A critique of the literature on parenting gifted learners. J. Educ. Gift.

[119] Smith S., Frydenberg E., Martin A., Collie R. (2017). Responding to the unique social and emotional learning needs of gifted Australian students. Social and Emotional Learning in Australia and the Asia-Pacific.

[120] Cheung C.K., Cheung H.Y., Hue M.T. (2014). Emotional intelligence as a basis for self-esteem in young adults. J. Psychol..

[121] Suldo S.M., Hearon B.V., Bander B., McCullough M., Garofano J., Roth R.A., Tan S.Y. (2015). Increasing elementary school students’ subjective well-being through a classwide positive psychology intervention: Results of a pilot study. Contemp. Sch. Psychol..

[122] Shoshani A., Slone M. (2017). Positive education for young children: Effects of a positive psychology intervention for preschool children on subjective well-being and learning behaviors. Front. Psychol..

[123] Castillo R., Fernández-Berrocal P., Brackett M.A. (2013). Enhancing teacher effectiveness in Spain: A pilot study of the RULER approach to social and emotional learning. J. Educ. Train. Stud..

[124] Conners-Burrow N.A., Patrick T., Kyzer A., McKelvey L. (2017). A preliminary evaluation of REACH: Training early childhood teachers to support children’s social and emotional development. Early Child. Educ. J..

[125] Nelson L.J., Rubin R.H., Fox N.A. (2005). Social withdrawal, observed peer acceptance, and the development of self-perceptions in children ages 4 to 7 years. Early Child. Res. Q..

